# Chloroquine-induced DNA damage synergizes with DNA repair inhibitors causing cancer cell death

**DOI:** 10.3389/fonc.2024.1390518

**Published:** 2024-05-13

**Authors:** Diego Iglesias-Corral, Paula García-Valles, Nuria Arroyo-Garrapucho, Elena Bueno-Martínez, Juan Manuel Ruiz-Robles, María Ovejero-Sánchez, Rogelio González-Sarmiento, Ana Belén Herrero

**Affiliations:** ^1^Institute of Biomedical Research of Salamanca (IBSAL), Salamanca, Spain; ^2^Molecular Medicine Unit, Department of Medicine, University of Salamanca, Salamanca, Spain; ^3^Institute of Molecular and Cellular Biology of Cancer (IBMCC), University of Salamanca-CSIC, Salamanca, Spain

**Keywords:** breast cancer, colorectal cancer, head and neck cancer, glioblastoma, chloroquine, DNA repair inhibitors, drug combination

## Abstract

**Background:**

Cancer is a global health problem accounting for nearly one in six deaths worldwide. Conventional treatments together with new therapies have increased survival to this devastating disease. However, the persistent challenges of treatment resistance and the limited therapeutic arsenal available for specific cancer types still make research in new therapeutic strategies an urgent need.

**Methods:**

Chloroquine was tested in combination with different drugs (Panobinostat, KU-57788 and NU-7026) in 8 human-derived cancer cells lines (colorectal: HCT116 and HT29; breast: MDA-MB-231 and HCC1937; glioblastoma: A-172 and LN-18; head and neck: CAL-33 and 32816). Drug´s effect on proliferation was tested by MTT assays and cell death was assessed by Anexin V-PI apoptosis assays. The presence of DNA double-strand breaks was analyzed by phospho-H2AX fluorescent staining. To measure homologous recombination efficiency the HR-GFP reporter was used, which allows flow cytometry-based detection of HR stimulated by I-SceI endonuclease-induced DSBs.

**Results:**

The combination of chloroquine with any of the drugs employed displayed potent synergistic effects on apoptosis induction, with particularly pronounced efficacy observed in glioblastoma and head and neck cancer cell lines. We found that chloroquine produced DNA double strand breaks that depended on reactive oxygen species formation, whereas Panobinostat inhibited DNA double-strand breaks repair by homologous recombination. Cell death caused by chloroquine/Panobinostat combination were significantly reduced by N-Acetylcysteine, a reactive oxygen species scavenger, underscoring the pivotal role of DSB generation in CQ/LBH-induced lethality. Based on these data, we also explored the combination of CQ with KU-57788 and NU-7026, two inhibitors of the other main DSB repair pathway, nonhomologous end joining (NHEJ), and again synergistic effects on apoptosis induction were observed.

**Conclusion:**

Our data provide a rationale for the clinical investigation of CQ in combination with DSB inhibitors for the treatment of different solid tumors.

## Introduction

Cancer stands as a major health issue worldwide; the number of new cancer cases diagnosed in 2020 was 19.3 million, and almost 10.0 million people die every year due to this disease (1). Improvements in the conventional treatments, which include surgery, radiation therapy, and chemotherapy together with new approaches, such as immune-mediated therapies or targeted therapy are increasing survival rates of this devastating disease. However, the emergence of resistances to all these treatments remains one of the biggest challenges in the field of cancer research. One potential way to reduce resistances is through the combination of anticancer therapies (2). Additionally, combined drug regimens present several other advantages, such as the reduction of toxicity, which allows using individual drugs at lower dosages while maintaining therapeutic efficacy, particularly when a synergistic anticancer activity is achieved. Our group have recently reported that the combination of chloroquine (CQ) with a histone deacetylase inhibitor, Panobinostat (LBH), or with inhibitors of the nonhomologous end joining (NHEJ) DNA repair pathway, resulted in significant synergistic effects in ovarian cancer cell lines (3, 4). Therefore, it is of interest to analyze and compare the effect of these combinations in other tumor types.

CQ is a well-known autophagy inhibitor initially discovered and employed for the prevention and treatment of malaria (5). This compound has also been used as an anti-inflammatory agent to treat several inflammatory diseases (5, 6) and many reports highlight its prominent role as an anticancer agent. In fact, it is considered one of the most prominent instances of drug repurposing in cancer (7). CQ has been described to reduce hypoxia, cancer cell invasion and metastasis, while improving chemotherapy delivery and response (8, 9). Thus, several preclinical results and clinical trials have shown that CQ sensitizes tumor cells to radiotherapy or chemotherapy (6, 8, 10–22). The anticancer effect of CQ has been mainly attributed to its ability to inhibit autophagy (6, 10, 11, 13, 18, 22), which serves as a resistance mechanism against chemotherapy (23). However, other autophagy-independent antitumoral effects have also been described (8, 19, 24–27). In this regard, we have recently published that CQ increases reactive oxygen species (ROS) in ovarian cancer cell lines, causing DNA double-strand breaks (DSBs) (3, 4), the most lethal form of DNA damage (28).

Histone deacetylase inhibitors (HDACi) also represent promising agents in cancer treatment, particularly in combination with other anti-cancer drugs and/or radiotherapy (29–31). These molecules inhibit HDACs, enzymes responsible for removing acetyl groups from lysine residues thereby acting as transcriptional repressors (32). The inhibition of these enzymes promotes transcriptional activation of multiple genes that are typically silenced in human tumors (33). Moreover, HDACi have also been shown to exert pleiotropic antitumor effects; they induce the expression of proapoptotic genes, cause cellular differentiation and/or cell cycle arrest (29, 34, 35). That is the case of Panobinostat (LBH), an HDACi approved in 2015 for the treatment of different hematological malignancies (30). HDACi and LBH also act as autophagy inducers, but this effect is not considered and antitumor mechanism but rather a potential mechanism of resistance. For this reason, some researchers have explored the combination of HDACi with autophagy inhibitors, discovering synergistic effects in several tumor cell lines, such as breast, colon, leukemic and neuroblastoma cell lines (10–13, 18, 22). However, our prior research indicated that the cell death induced by the CQ/LBH combination in ovarian cancer cells was largely dependent on DSB induction by CQ and the HR inhibition caused by LBH (3). These effects have not yet been investigated in other types of tumors.

Our group has also recently published findings indicating that CQ-induced DNA damage synergizes with nonhomologous end joining (NHEJ) inhibition, resulting in cytotoxicity in ovarian cancer cells (4). Due to the inhibition of DSB repair, NHEJ inhibitors (NHEJi) have been shown to increase the cytotoxicity of several genotoxic drugs or radiotherapy in different cancer types (36–41). However, the combination of CQ with these compounds has neither been explored in other types of tumors.

In this study, we analyzed the effect of CQ, LBH and NHEJi, both individually or in combination, in four different tumor types, two with very high incidence: breast and colorectal cancer, and two with limited approved treatments and poor outcomes: head and neck cancer and glioblastoma. Our findings reveal that CQ induces DNA double strand breaks (DSBs) in all the cell lines analyzed, which largely depends on ROS production. Consequently, combination of CQ with NHEJi or with LBH, which we demonstrate inhibits DSB repair by HR, elicits a strong synergistic effect that should be explored in *in vivo* studies and subsequent in clinical trials.

## Materials and methods

### Cell lines and culture conditions

The following human cell lines were used: colon cancer HCT116 (RRID: CVCL_0291) and HT29 (RRID: CVCL_0320), breast cancer MDA-MB-231 (RRID: CVCL_0062) and HCC1937 (RRID: CVCL_0290), glioblastoma A-172 (RRID: CVCL_0131) and LN-18 (RRID: CVCL_0392) and head and neck cancer CAL-33 (ACC 447) and 32816. All of them were acquired from the American Type Culture Collection (ATCC), with the exception of CAL-33, obtained from the DSMZ German Collection of Microorganisms and 32816 which was established in our laboratory from an oropharyngeal squamous cell carcinoma.

Colon and glioblastoma cancer cell lines and the breast cancer cell line MDA-MD-231 were cultured in Dulbecco’s modified Eagle’s medium (DMEM) (Gibco, Waltham, MA, USA), whereas breast cancer cell line HCC1937, and head and neck cancer cell lines were cultured in RPMI 1640 medium (Gibco, Waltham, MA, USA). Both types of media were supplemented with 10% FBS and 1% penicillin/streptomycin. All cells were incubated at 37°C in a 5% CO2 atmosphere. The presence of mycoplasma was routinely checked using the MycoAlert kit (Lonza, Basel, Switzerland) and only mycoplasma-free cells were employed in the experiments.

### Reagents

Chloroquine (CQ) and N-Acetylcysteine (NAC) were purchased from Sigma-Aldrich (St. Louis, MO, USA). KU-57788 (KU), NU-7026 (NU) and Mirin were obtained from MedChemExpress (South Brunswick Township, NJ, USA), and Panobinostat (LBH) was provided by Novartis Pharmaceuticals, Basel, Switzerland.

### Cell proliferation assay

Cell lines were seeded into 96-well plates (from 2,000 to 4,000 cells/well, depending on the cell lines used) and were treated with different concentrations of CQ, LBH, KU-57788 or NU-7026 for 24, 48 or 72 h. Cell proliferation was determined using 3-(4,5-dimethylthiazol-2-yl)- 2,5-diphenyl-2H-tetrazolium bromide (MTT) (Sigma-Aldrich). MTT was dissolved in PBS at 5 mg/mL and 10 μL of the solution was added to each well. After 1 h of incubation, medium was aspirated, and formazan crystals were dissolved in DMSO (100 μL/well). Absorbance was measured in a plate reader (Ultra Evolution, Tecan) at 570 nm. The half maximal inhibitory concentration (IC50) was calculated using GraphPad Prism 8 (RRID: SCR_002798).

### Apoptosis assay

Cancer cell lines were treated with the different drugs for 72 h then stained with FITC Apoptosis Detection Kit CE (Immunostep, Salamanca, Spain) according to the manufacturer’s guidelines. The percentage of apoptotic cells was determined by flow cytometry using a BD Accuri C6 Plus Flow Cytometer. The synergism of the combination was determined using CompuSyn Software (version 1.0 for Windows, ComboSyn, Inc., Paramus, NJ, USA), based on the Chou-Talalay method (42), which calculates the combination index (CI) with the following interpretation: CI > 1: antagonistic effect; CI = 1: additive effect; CI < 1 synergistic effect.

### Immunofluorescence

Cancer cell lines were plated on round glass coverslips (12 mm diameter) (200,000 cells/well in 6-well plates) and, after 24 h of culture, cells were treated with CQ or CQ+NAC for 48 h. Then, cells were fixed with 4% paraformaldehyde for 10 min, permeabilized with 0.5% Triton X-100 (Boehringer Mannheim) in PBS for 10 min, blocked in 10% BSA in PBS for 30 min and incubated with phospho-H2AX antibody (1:1000, Sigma-Aldrich, RRID: AB_309864) for 90 min. After washing, coverslips were incubated with fluorescent secondary antibodies (1:400, Alexa Fluor 488 goat anti-mouse IgG, RRID: AB_141607) for 1 h. DAPI (dihydrochloride of 4’, 6-diamidino-2-phenylindole, Roche) was used to visualize the nuclei. Mowiol reagent (Calbiochem, San Diego, CA, USA) was used to fix preparations on slides. Cells were then analyzed by confocal microscopy (63x) using a LEICA SP5 microscope DMI-6000V model coupled to a LEICA LAS AF software computer.

### Homologous recombination functional assay

HCT116, HT29, HCC1937, A-172, LN-18, CAL-33 and 32816 cancer cell lines were transfected with 1 μg of pHR plasmid, kindly provided by Dr Gorbunova (43) linearized by digestion with the restriction enzyme NheI. G418 was added at 500 μg/mL 72 h post- transfection and stable pools were obtained after 3 weeks of selection. To measure HR efficiency in stable pools, cells were first preincubated with LBH for 24 h. Then, 10**^6^** cells were cotransfected with 5 μg of a plasmid that express the endonuclease I-SceI and 0.5 μg of pDsRed-N1 (Clontech, Palo Alto, CA, USA, Cat.632429) to correct for differences in transfection efficiencies. Transfections were performed using the Amaxa Cell Line Nucleofector Kit V and Amaxa Nucleofector device (Lonza). Programs used were D-032 for HCT116, W-017 for HT29, A-023 for HCC1937, T-016 for A-172 and X-001 for LN-18. After transfection, cells were incubated again with the same concentration of Panobinostat for 48 h. Live cells were selected by FSC/SSC gating, and live GFP+ and DsRed+ cells were quantified by flow cytometry (BD Accuri C6 Plus Flow Cytometer). HR efficiency was calculated as the ratio of GFP + to DsRed + cells.

### Statistical analysis

Differences between the results obtained from treated and nontreated cells were assessed for statistical significance using Student’s unpaired 2 tailed t-test with IBM SPSS Statistics for Windows version 25.0 (IBM Corporation, RRID: SCR_016479). Data are presented as mean ± standard deviations. Statistical significance was concluded for values of p ≤0.05.

## Results

### Chloroquine inhibits proliferation of colon, breast, glioblastoma and head and neck cancer cell lines

**Figure 1:**
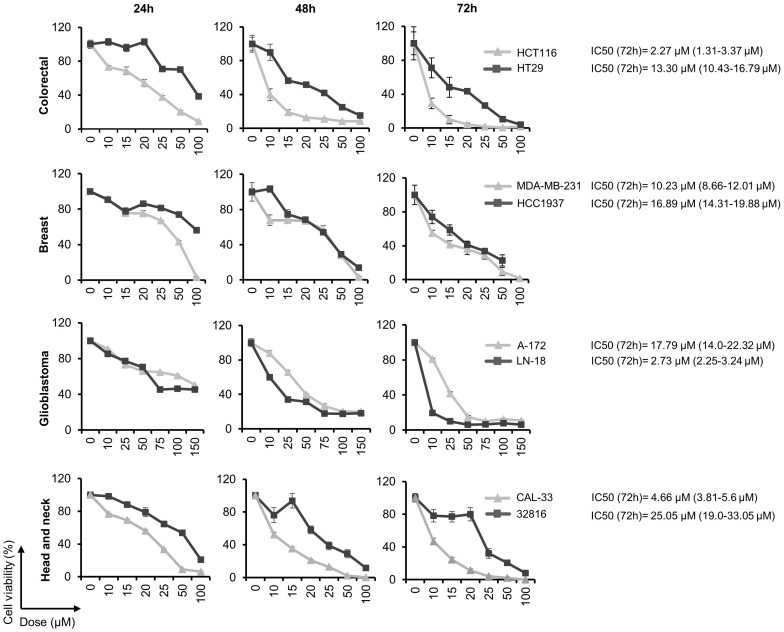
Chloroquine inhibits cell proliferation in a variety of human solid tumor cell lines. Cell viability was calculated by MTT assays after treatment with the indicated doses of CQ for 24, 48 and 72 h. The half maximal inhibitory concentration (IC50) was calculated at 72 h post-treatment.

The effect of CQ on the tumor cell lines proliferation was evaluated at different time points and concentrations by MTT assays. We found that treatment with this drug inhibited cell proliferation in a dose- and time-dependent manner in all the cell lines analyzed (**Figure 1**). IC50 values, calculated at 72 hours post-treatment, ranged from 2.27 μM in the colon cell line HCT116 to 25.05 in 32816, a head and neck cancer cell line established in our laboratory.

### Chloroquine induces DSBs in cancer cell lines that are avoided by the addition of N-acetylcysteine

**Figure 2:**
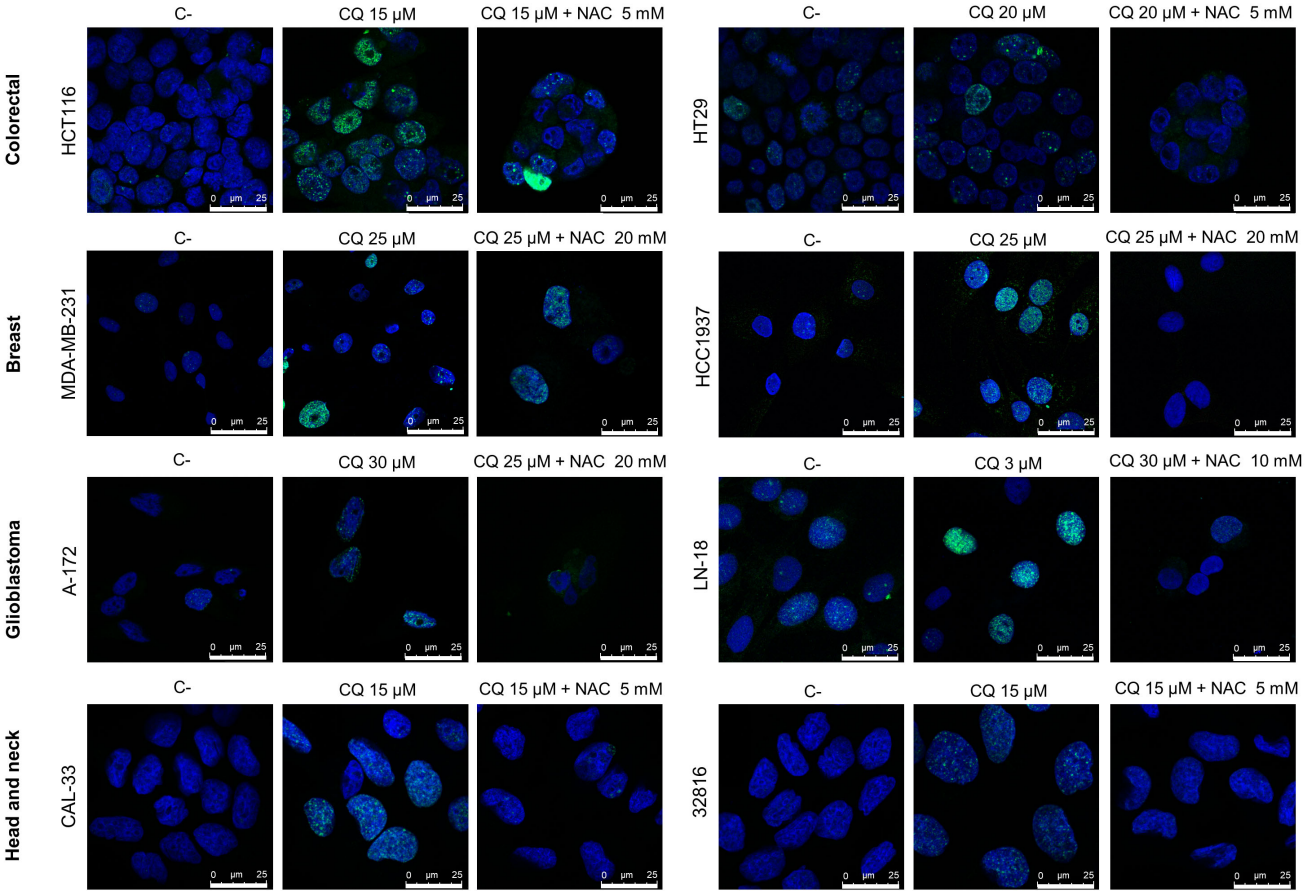
Chloroquine induces DNA double-strand-breaks (DSBs) in cancer cell lines that are prevented by the addition of N-Acetylcysteine (NAC). Cells were treated with CQ for 48 h in the presence or absence of NAC, then fixed with paraformaldehyde, incubated with phospho-H2AX antibodies and stained with DAPI. γH2AX foci were visualized by confocal microscopy.

CQ has been demonstrated to exert pleiotropic cellular effects, including the generation of reactive oxygen species (ROS) (44–47). We have recently shown that CQ-induced ROS production in ovarian cancer cell lines leads to the generation of DNA-DSBs (3). To analyze whether CQ also induced these lethal lesions in other cancer cell lines we treated colon, breast, glioblastoma and head and neck cancer cell lines with this compound and then monitored the phosphorylation of H2AX (γH2AX), a sensitive and well-recognized marker of DSBs, by immunofluorescence. CQ was found to induce DSBs in all the cell lines analyzed (**Figure 2**). Moreover, addition of the antioxidant NAC decreased the number of cells with γH2AX foci, which clearly indicate that CQ-induced DSBs are caused by ROS.

### Panobinostat inhibits proliferation of colon, breast, glioblastoma and head and neck cancer cell lines

**Figure 3:**
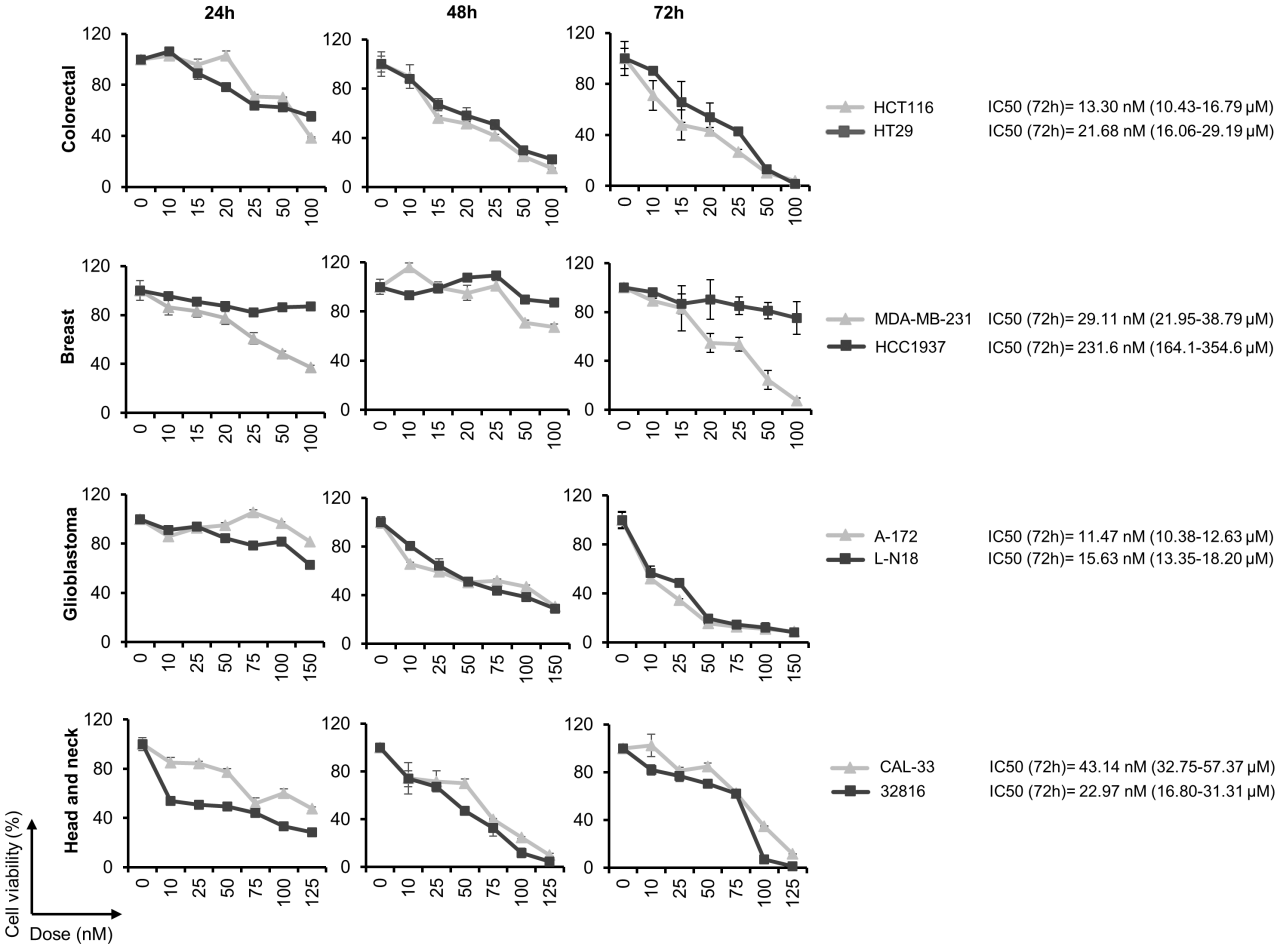
Panobinostat inhibits cell proliferation in a variety of human solid tumor cell lines. Cell viability was calculated by the MTT assay after treatment with the indicated doses of LBH for 24, 48 and 72 h. The half maximal inhibitory concentration (IC50) was calculated at 72 h post-treatment.

Next, we studied the effect of Panobinostat (LBH) on the proliferation of the different cancer cell lines. A dose- and time-dependent effect was observed in all the cell lines analyzed (**Figure 3**), with the lowest IC50s observed in the glioblastoma cell lines (11.47 nM in A-172 and 15.6 nM in LN-18) and the highest in the breast cancer cell line HCC1937 (231.6 nM).

### Panobinostat inhibits homologous recombination repair in cancer cell lines

**Figure 4:**
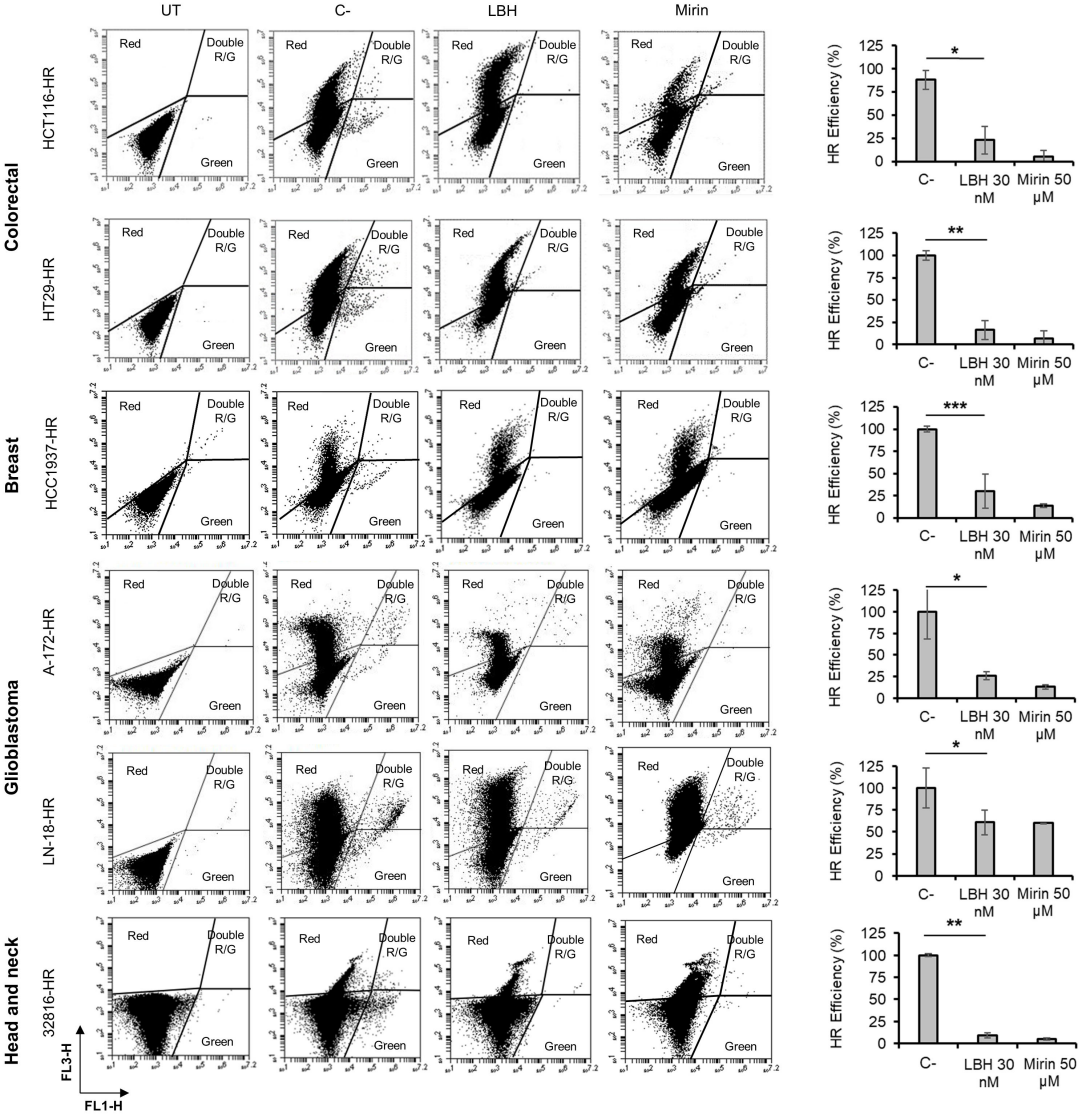
Panobinostat inhibits DSB repair by homologous recombination. Tumor cell lines carrying the HR reporter cassette were pre-treated or not (C-) with LBH for 24 h and then co-transfected with 5 μg of an I-SceI endonuclease-expressing plasmid and 0.5 μg of pDsRed2-N1. UT correspond to untreated and non-transfected cells. Cells were then incubated in the presence or absence (C-) of LBH or Mirin for additional 48 h. Correct HR repair restored GFP gene and was detected as green cells. HR efficiency was calculated as the ratio of GFP + to DsRed + cells. Histograms show the mean of 3 independent experiments. Error bars represent the SD (**P < 0.01, *P < 0.05; ***P < 0.001).

It has been described that some HDACi decrease DNA DSB repair by inhibiting homologous recombination (HR) (48, 49). Moreover, we previously reported a significant reduction in HR efficiency after treatment of ovarian cancer cell lines with Panobinostat (3). To analyze the effect of this drug in the repair ability of the tumor types under study, we first created stable cell lines carrying a chromosomally integrated GFP-based reporter cassette. In this system, correct repair by HR of DSBs induced by an endonuclease restored a functional GFP that is detectable by flow cytometry (green cells). Colon, breast, glioblastoma and head and neck cells carrying the HR cassette were pretreated with LBH for 24 h and then transfected with an I-SceI endonuclease-expressing plasmid together with the pDsRed-N1 plasmid (red), to normalize for transfection efficiency, then incubated again for an additional 48 h. Mirin, an inhibitor of MRN complex (Mre11-Rad51-Nsb1) required for HR, was used as a control (50). In all the cell lines analyzed, we found a significant reduction in the number of HR-proficient cells that had been treated with LBH compared with untreated cells (**Figure 4**). These results reveal a clear defect in the HR mechanism in the presence of this HDACi.

### Combination of chloroquine and LBH synergistically induces cell death in colon, breast, glioblastoma and head and neck cancer cell lines

**Figure 5:**
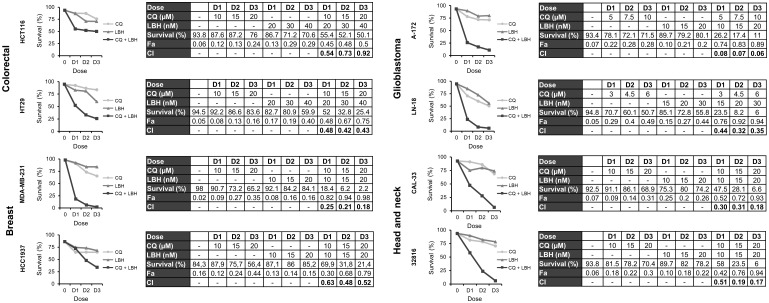
Synergistic effect of chloroquine and Panobinostat in the different cancer cell lines. Cells were treated for 72 h with the indicated concentrations of LBH and CQ at a constant ratio and survival was assessed by flow cytometry after staining with Annexin-/PI-. CI values less than 1 indicated a synergistic effect. These values were calculated using Compusyn Software.

Once we established that CQ induced DNA damage and LBH reduced DSB repair by HR in all the cell lines under study, we analyzed the effect of combining both drugs on apoptosis induction. As shown in **Supplementary Figure S1** CQ and LBH induced apoptosis when used individually, but the percentage of apoptotic cells was much higher when these compounds were combined in all the cell lines analyzed. To determine the type of interaction between CQ and LBH, the combination indices (CIs) at three different drug doses were calculated using CompuSyn software (**Figure 5**). In all cases, CIs were below 1, indicating synergistic interactions. The cell line A-172 was the one with the lowest CI (0.06), despite of using lower CQ doses than in the rest of the cell lines assayed. CIs were also very low in the breast cell line MDA-MB-231 and in both head and neck cancer cell lines.

### Reactive oxygen species generation plays an important role in CQ/LBH-induced lethality

**Figure 6:**
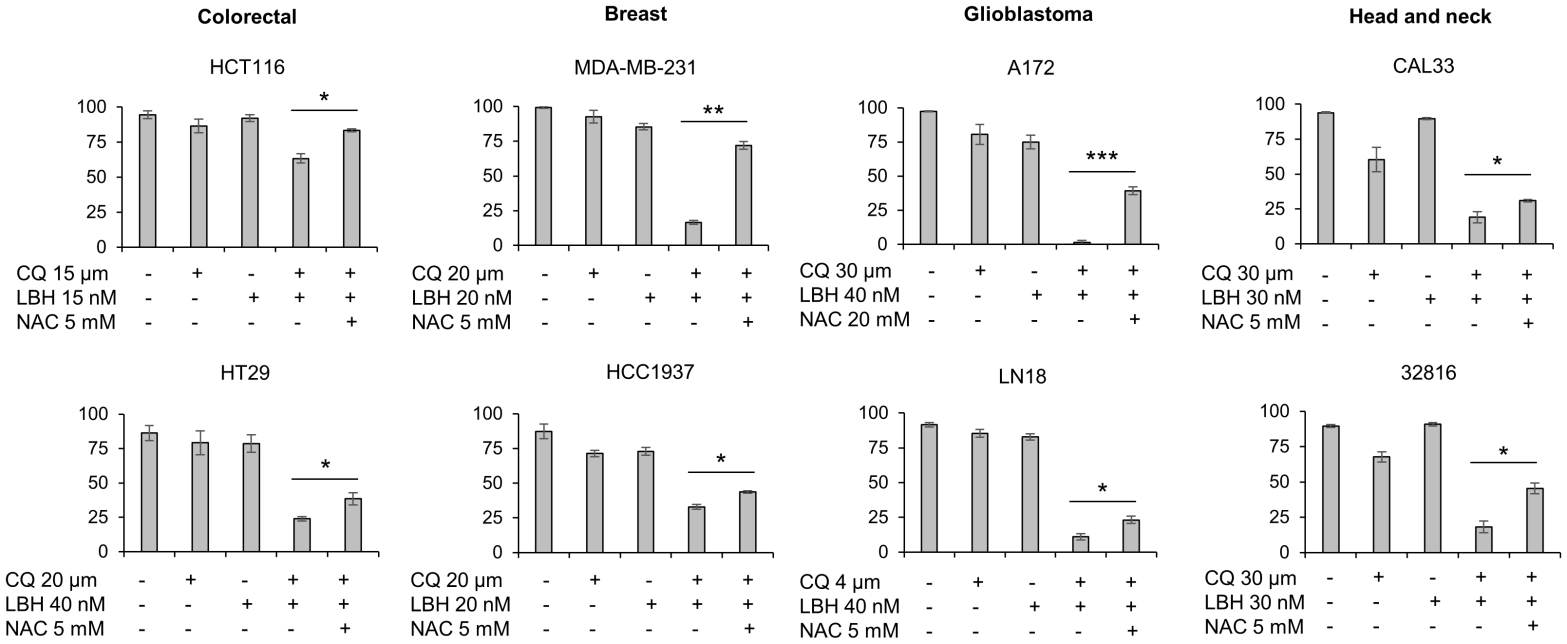
Cell death caused by the combination of chloroquine and LBH depends on ROS production. Cells were exposed for 72 h to the indicated concentrations of CQ (µM), LBH (nM) and the ROS scavenger NAC (mM) and the percentage of apoptotic cells were measured after cell staining with annexin V and propidium iodide by flow cytometry. Data are the mean of at least 3 independent experiments. Error bars represent the SD (***p < 0.001, **p < 0.01, and *p < 0.05).

We previously reported that CQ/LBH-induced lethality in ovarian cancer cell lines largely depended on DSBs caused by ROS. To analyze whether ROS generation was also crucial in triggering apoptotic cell death in other tumor types, the different cell lines were treated with CQ, LBH or their combination in the presence or absence of N-acetylcysteine. Cell survival was analyzed after 72 h of incubation by Annexin/PI staining. As shown in **Figure 6**, adding the antioxidant significantly prevented cell death caused by the combination of CQ and LBH in all the cancer cell lines analyzed, being the glioblastoma cell line A-172 the one where the antioxidant protected the most against CQ/LBH-induced cell death.

### NHEJi decrease proliferation rates in colon, breast, glioblastoma and head and neck cancer cell lines

We have previously reported that treatment with NHEJi decreased cell proliferation in ovarian cancer cell lines (4). To determine whether NHEJi also affected the growth of other tumor types, we performed MTT assays using different concentrations of the known NHEJi KU-57788 and NU-7026 (**Supplementary Figures S2****,** **S3**). We found that both compounds inhibited cell proliferation in a dose-dependent manner in all cell lines analyzed. Inhibition of cell proliferation was similar at the 3 times analyzed in most of the cell lines, revealing a quick and sustained effect. The colon cancer cell line HCT116 was the most affected by both NHEJi, with IC50s of 4.52 µM for NU-7026 and 1.88 µM for KU-57788.

### Combination of chloroquine and NHEJi synergistically induces cell death in colon, breast, glioblastoma and head and neck cancer cell lines

**Figure 7:**
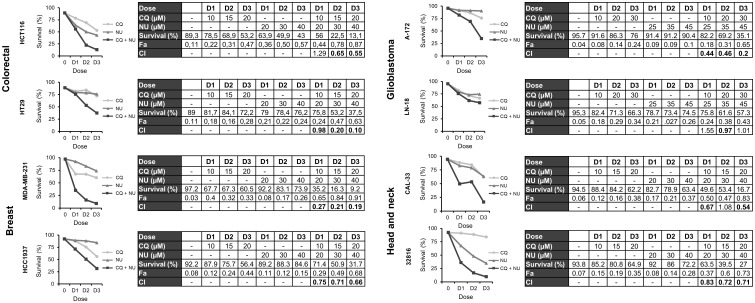
Synergistic effect of chloroquine and NU-7026 in various cancer cell lines. Cells were treated for 72 h with the indicated concentrations of CQ and NU-7026 at a constant ratio and survival was assessed by flow cytometry after staining with Annexin-/PI-. CI values less than 1 indicated a synergistic effect. These values were calculated using Compusyn Software.

**Figure 8:**
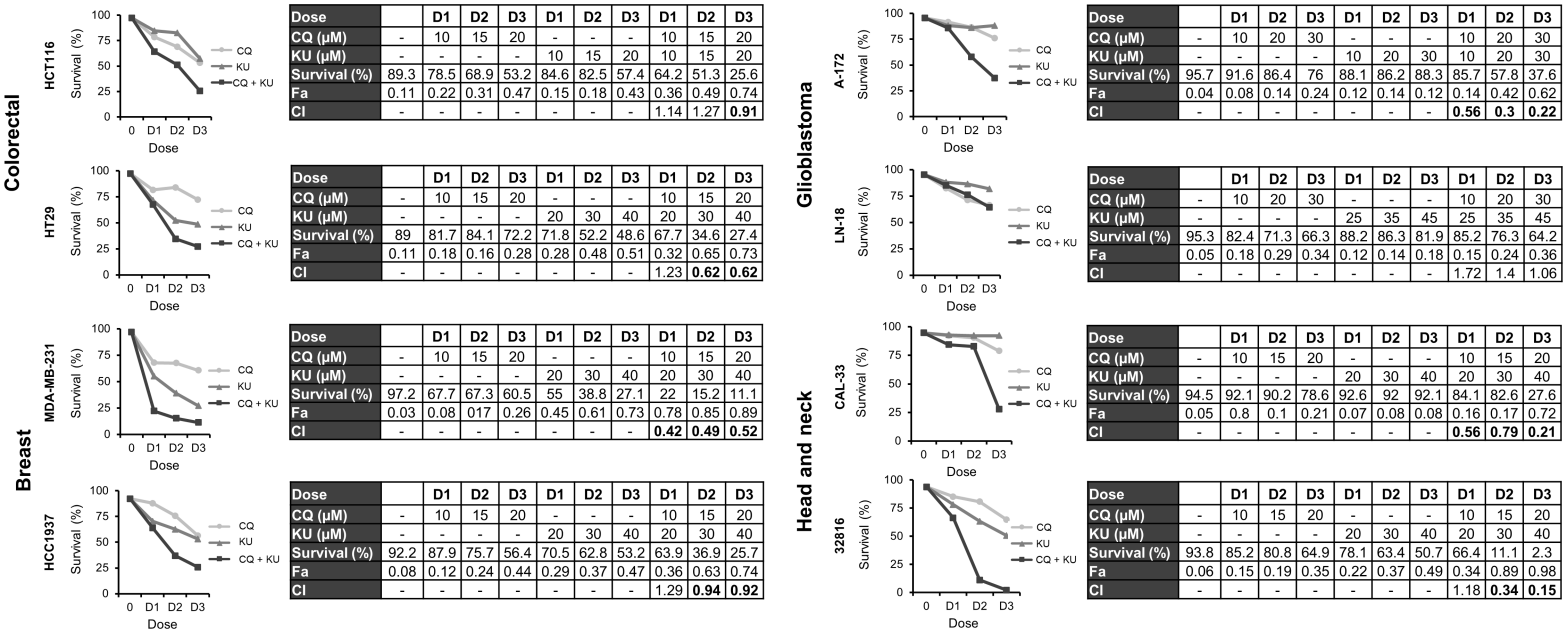
Synergistic effect of chloroquine and KU-57788 in in various cancer cell lines. Cells were treated for 72 h with the indicated concentrations of CQ and KU-57788 at a constant ratio and survival was assessed by flow cytometry after staining with Annexin-/PI-. CI values less than 1 indicated a synergistic effect. These values were calculated using Compusyn Software.

Finally, we were interested in determining whether the combination of CQ with NHEJi was also effective in the different tumor cell lines under study. First, we monitored apoptosis induction after treatment with CQ, NU-7026 or KU-57788 and their combinations. As shown in **Supplementary Figures S4** and **S5**, Annexin+ cells were observed in all the cases, but the percentage of apoptotic cells was much higher when CQ was combined with either of the NHEJi. Then we assayed three different drug doses and calculated the CIs using the CompuSyn software. In all the cases, except in the glioblastoma cell line LN-18, CIs were below 1 indicating synergistic interactions (**Figures 7**, **8**).

## Discussion

The combination of two or more therapeutic agents is a cornerstone in cancer treatment. This approach is particularly interesting when synergistic interactions are achieved and especially when it involves repurposing drugs, as it would reduce timelines and cost compared with *de novo* drug development. In this study, we report, for the first time, that the combination of the antimalarial drug CQ with the HDACi LBH or with NHEJi produces a robust synergistic effect in breast, colon, glioblastoma and head and neck cancer cell lines. We demonstrate that CQ induces DNA DSBs dependent on ROS production and also that the repair of these lesions is inhibited by LBH, which avoids HR, or by NU-7026 and KU-57788, that inhibit the NHEJ repair pathway, which explains the observed synergies.

CQ has been widely used as an antimalarial and anti‐inflammatory agent for decades without any major side effects. This, and its promising preclinical results against several cancers, make this compound an outstanding candidate for drug repurposing in oncology (6, 16, 21). However, systematic studies comparing the response to this drug in different cancer types had not been conducted and are crucial to define its use in cancer treatment. Here, we found that CQ inhibited cell proliferation in colorectal, breast, glioblastoma and head and neck cancer cell lines, consistent with previous reports (51–53) although IC50s varied among the different cell lines (ranging from 2 to 25 µM). The underlying cause of this variability is challenging to identify, given the compound’s pleiotropic effects, which may vary depending on genetic backgrounds. One such effect is the production of ROS, that we previously observed in ovarian cancer cell lines (3) and have also been reported in other tumor types (44–47). CQ-induced ROS production directly caused DSBs, as demonstrated by cotreatment with the ROS scavenger NAC that prevented the appearance of these DNA lesions in ovarian cancer cell lines (3) and also in all the tumor cell lines used in this study. Some reports have described that CQ-induced oxidative damage activated the p53 pathway and induced apoptosis (E. L. 53, 54), so it is tempting to hypothesize that sensitivity to CQ might correlate with p53 status. However, although this seem to be the case in the colorectal cancer cell lines used in this study (p53 wild-type HCT116 was more sensitive than p53 mutated HT29), it does not hold true in glioblastoma, where the p53 wild-type A-172 cell line was more resistant than the p53 mutated LN-18.

Treatment with the HDACi LBH also produced an antiproliferative effect in the various tumor types analyzed, in agreement with previous reports (55). Similar to CQ, histone deacetylases inhibitors (HDACi) exert pleiotropic effects; they induce cell death, cell cycle arrest, angiogenesis reduction, and modulation of the immune system in cancer cells (55). Here, we demonstrate that LBH significantly reduces HR efficiency in all the cell lines tested, supporting its combination not only with CQ, but also with other therapies that produce DSBs directly or indirectly, such as radiation, cisplatin, bleomycin, doxorubicin or etoposide. In fact, previous studies have shown a synergistic effect with HDAC inhibitors and many of these treatments (56–58) with several combinations reaching clinical trials (30). We show a robust synergistic effect with the combination of CQ and LBH in all tested cell lines, particularly in glioblastoma, one of the most aggressive human cancers, and head and neck cancer cell lines, another cancer type with limited approved treatments and poor outcomes. The efficacy of this combination had previously been reported only in a triple negative breast cancer cell line (12) and in ovarian cancers (3). Interestingly, both CQ and also LBH have been shown to cross the blood-brain barrier (BBB) (59, 60), increasing the interest in glioma treatment. The synergistic effect of CQ in combination with HDAC inhibitors has previously been attributed to their opposite effects on autophagy, a potential mechanism of resistance to chemotherapy (10–13, 18, 22). However, we demonstrate that ROS generation plays a critical role in CQ/LBH-induced lethality in the different tumor cell lines analyzed, especially in the breast cancer MDA-MB-231 and in the glioblastoma A-172 cell line. It is possible that both mechanisms, autophagy modulation and DNA damage/inhibition of DNA repair, contribute variably to the cytotoxicity of the combination in the different tumor cell lines.

It has been described that the DNA-PK inhibitors NU-7026 and KU-57788 sensitize cancer cells to ionizing radiation and other DNA-damaging agents due to their known inhibition of NHEJ (36, 38, 40, 61), the other main mechanism involved in DSB repair together with HR. We found that both inhibitors exert and antiproliferative effect in all the cell lines analyzed, and when combined with CQ a potent synergistic effect was observed, except in LN-18, one of the two glioblastoma cell lines studied. This cell line exhibited high levels of endogenous DNA damage, suggesting an intrinsic alteration in the NHEJ repair pathway that might not be further affected by NU-7026 or KU-57788.

CQ is efficient in preclinical trials, although it has been noted to encounter challenges traversing the cell membrane due to the acidic extracellular microenvironments prevalent in the tumors (16). Recent advances in nanomedicine offer an opportunity to overcome this limitation. In fact, CQ encapsulation within nanoparticles has been shown to enhance its antitumor efficacy, prolonging drug circulation and reducing systemic toxicity *in vivo* (19).

In conclusion, our *in vitro* results suggest that the combination of CQ with DNA repair inhibitors could represent new therapeutic strategies against different cancer types beyond ovarian cancer (3 and 4), encompassing triple negative breast cancer, glioblastoma and head and neck cancers, all characterized by dismal prognoses and limited chemotherapy alternatives. The potential therapeutic value of CQ (either free or encapsulated) in combination with different DNA repair inhibitors requires further investigation. Future research will be directed to test the toxicity and effectiveness of the combinations in *in vivo* studies using xenograft mouse models.

## Data availability statement

The raw data supporting the conclusions of this article will be made available by the corresponding author at anah@usal.es, without undue reservation.

## Ethics statement

Ethical approval was not required for the studies on humans in accordance with the local legislation and institutional requirements because only commercially available established cell lines were used. Ethical approval was not required for the studies on animals in accordance with the local legislation and institutional requirements because only commercially available established cell lines were used.

## Author contributions

DI-C: Investigation, Methodology, Validation, Writing – review & editing. PG-V: Investigation, Validation, Writing – review & editing. NA-G: Investigation, Validation, Writing – review & editing. EB-M: Investigation, Validation, Writing – review & editing. JR-R: Investigation, Validation, Writing – review & editing. MO-S: Methodology, Validation, Writing – review & editing. RG-S: Funding acquisition, Resources, Supervision, Writing – review & editing. AH: Conceptualization, Supervision, Writing – original draft, Writing – review & editing.
