# Supplementary figures and images for: Chloroquine-induced DNA damage synergizes with DNA repair inhibitors causing cancer cell death
Source: Front Oncol. 2024 May 13;14:1390518. doi: 10.3389/fonc.2024.1390518 (PMC11128598; doi:10.3389/fonc.2024.1390518)

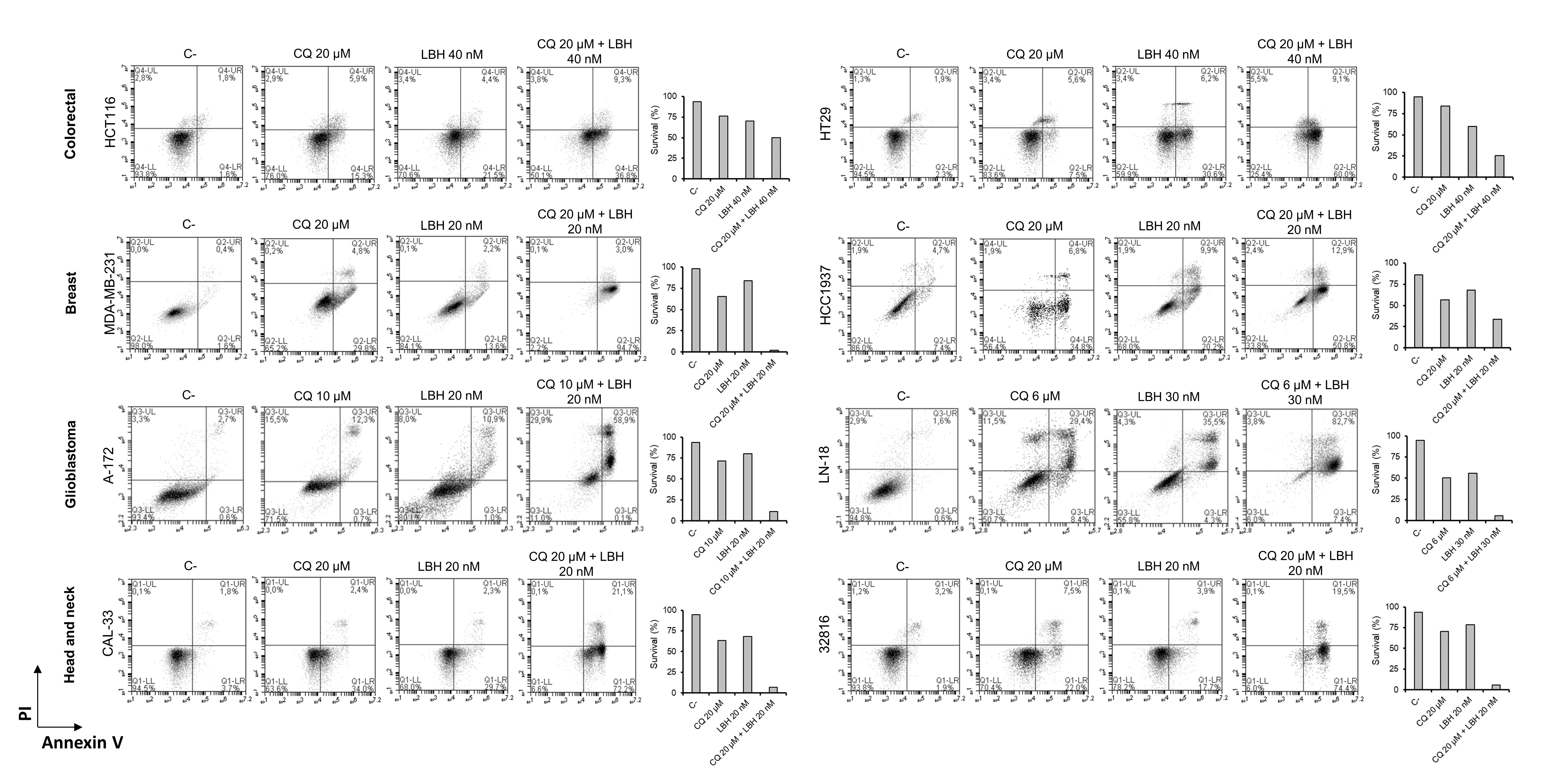

Supplement: Supplementary file 1 [file Image_1.tif]

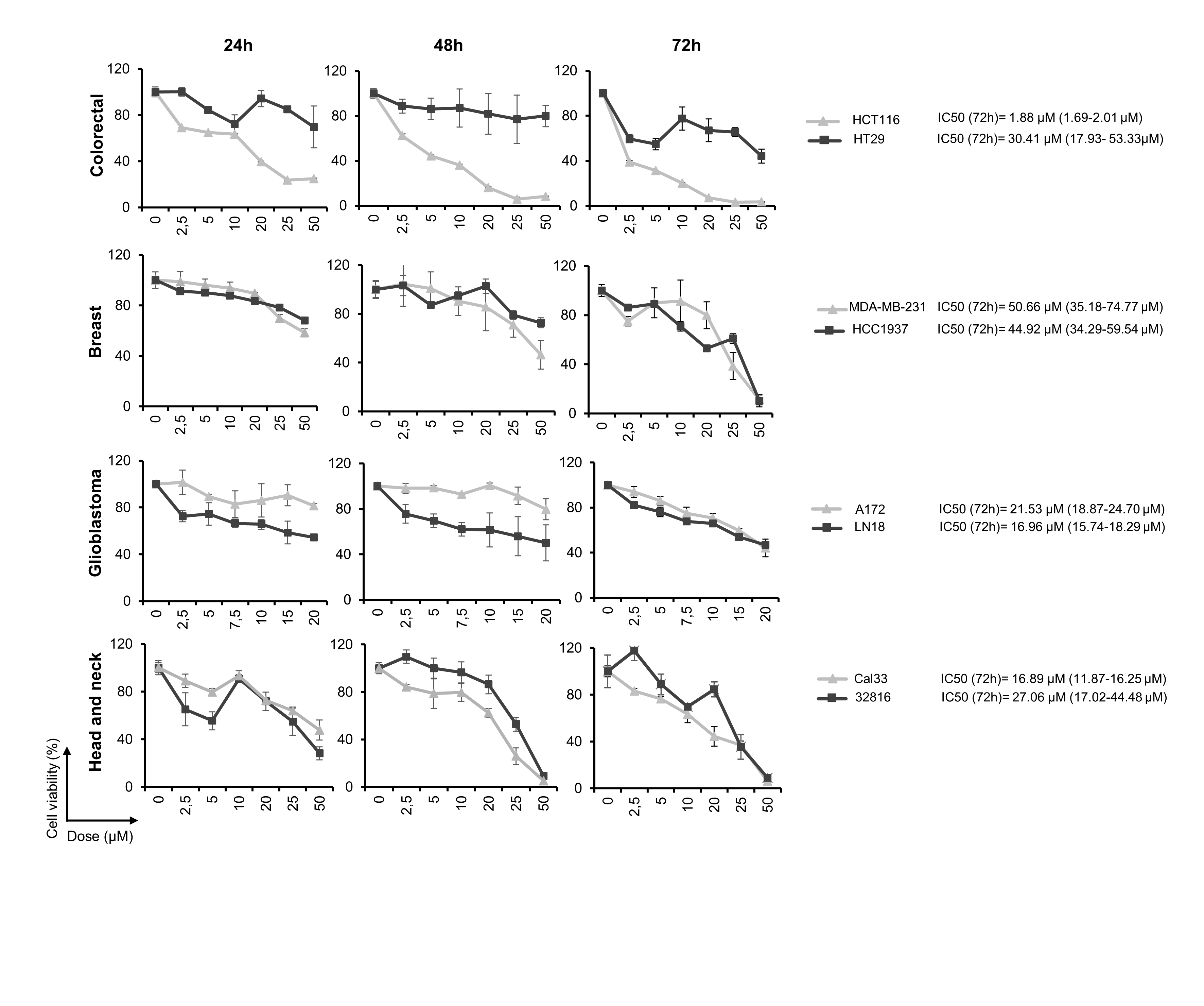

Supplement: Supplementary file 2 [file Image_2.tif]

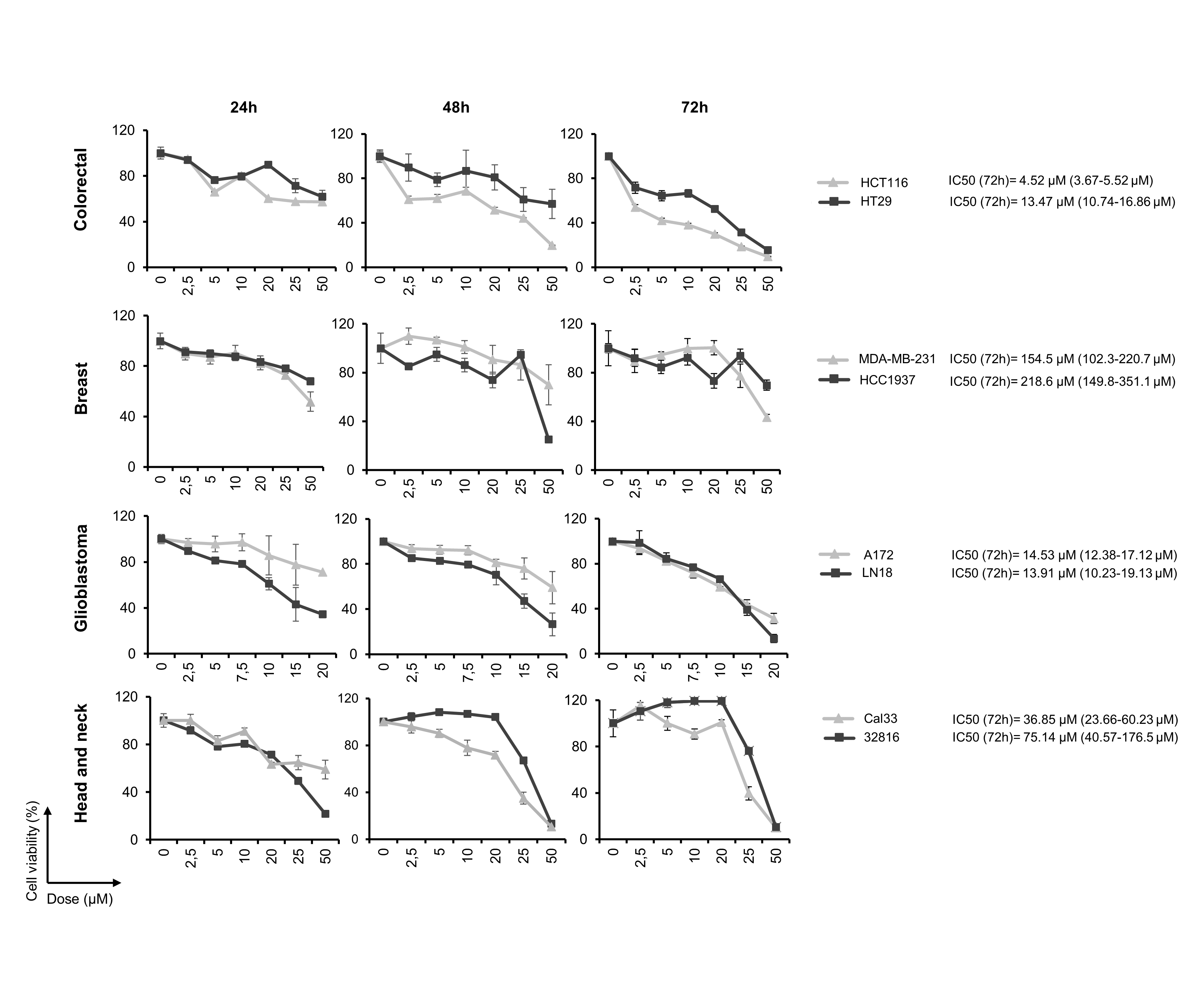

Supplement: Supplementary file 3 [file Image_3.tif]

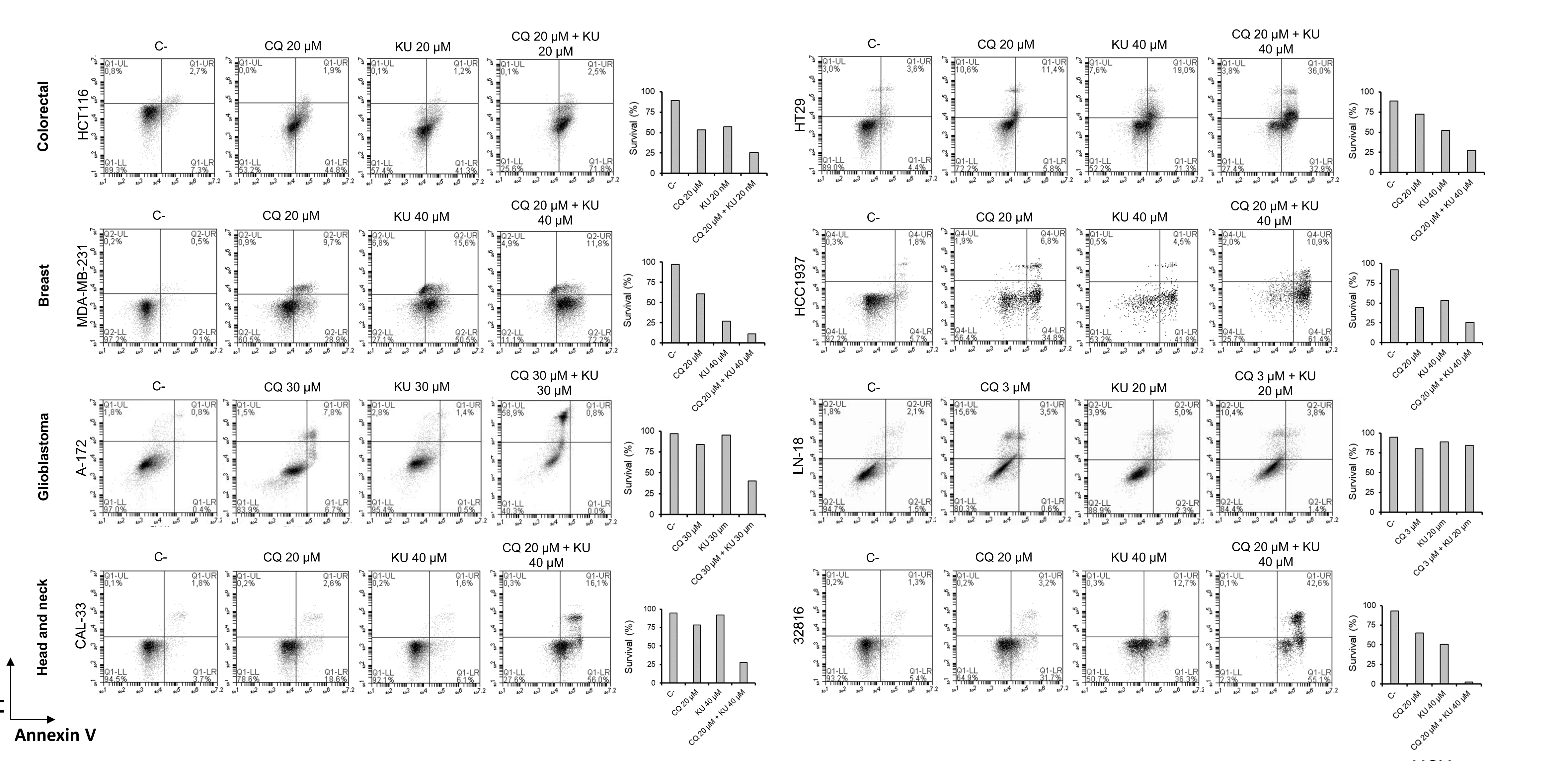

Supplement: Supplementary file 4 [file Image_4.tif]

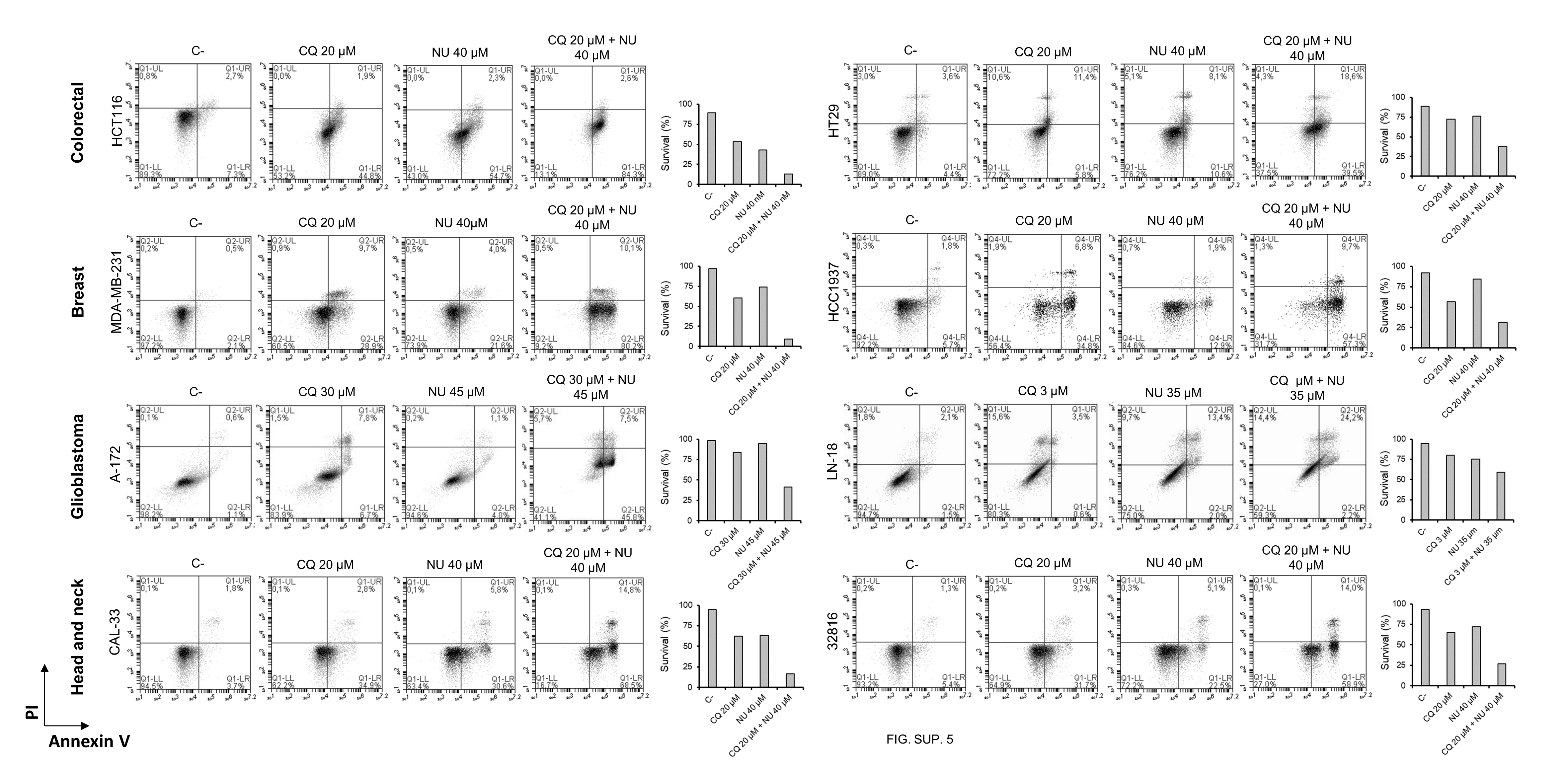

Supplement: Supplementary file 5 [file Image_5.tif]
